# Supplementary material for: Genome-Wide Analysis of Ribosomal Protein GhRPS6 and Its Role in Cotton Verticillium Wilt Resistance
Source: Int J Mol Sci. 2021 Feb 11;22(4):1795. doi: 10.3390/ijms22041795 (PMC7918698; doi:10.3390/ijms22041795)
Supplement: Supplementary file 1 [file ijms-22-01795-s001.pdf]

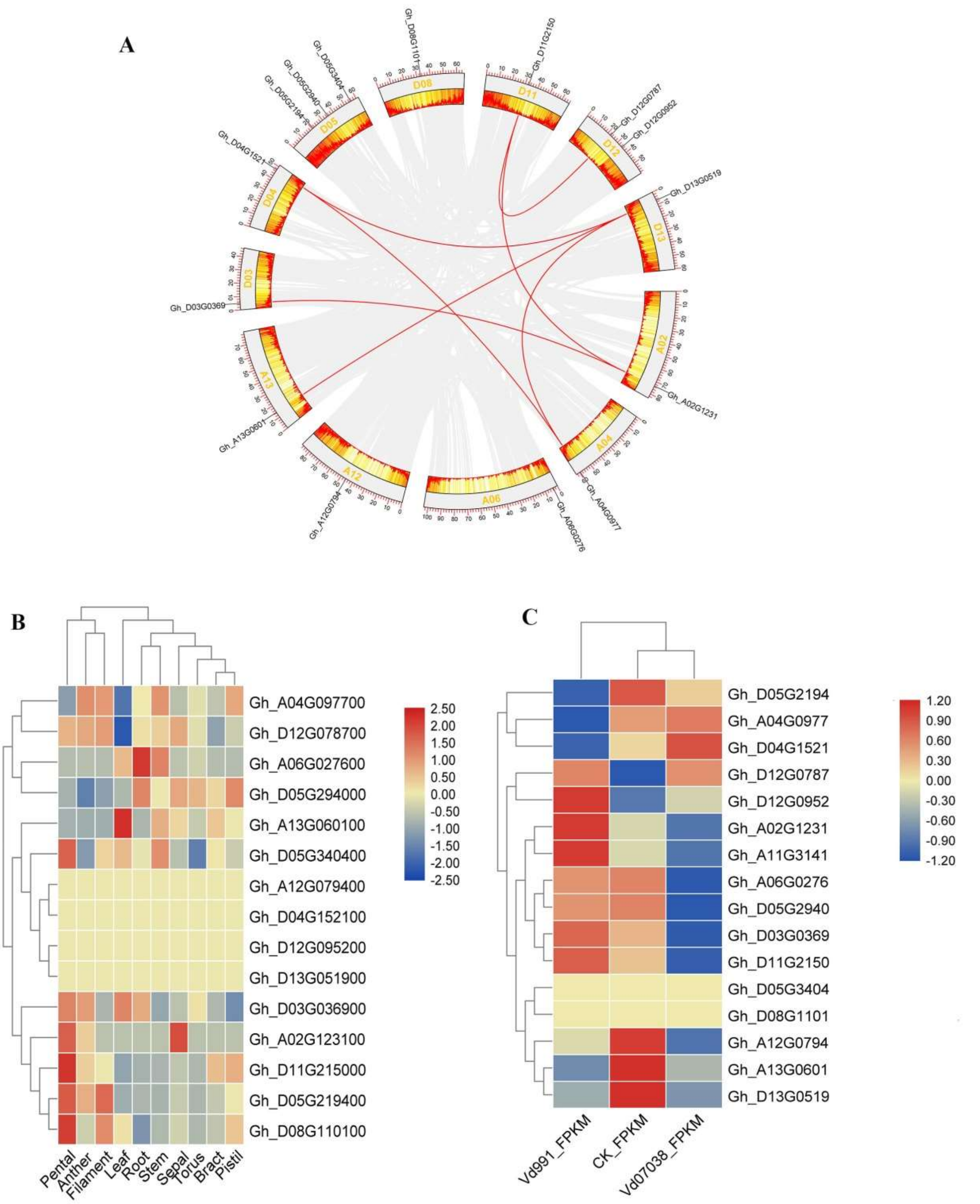

Supplementary Figure S1. *GhRPS6* chromosome location and expression profile. A, *GhRPS6* gene chromosome distribution and gene duplication. Genome visualization tool CIRCOS was used to illustrate the chromosome distribution of *GhRPS6* genes; B, The expression of *GhRPS6* in different tissues; C, *GhRPS6* gene expression in cotton root after different treatments. Vd991 is a highly pathogenic deciduous strain of *V. dahliae*, Vd07083 is the attenuated strain of *V. dahliae* and CK is the control of water. Transcriptome data are from our laboratory, NCBI login number is SRX12210.

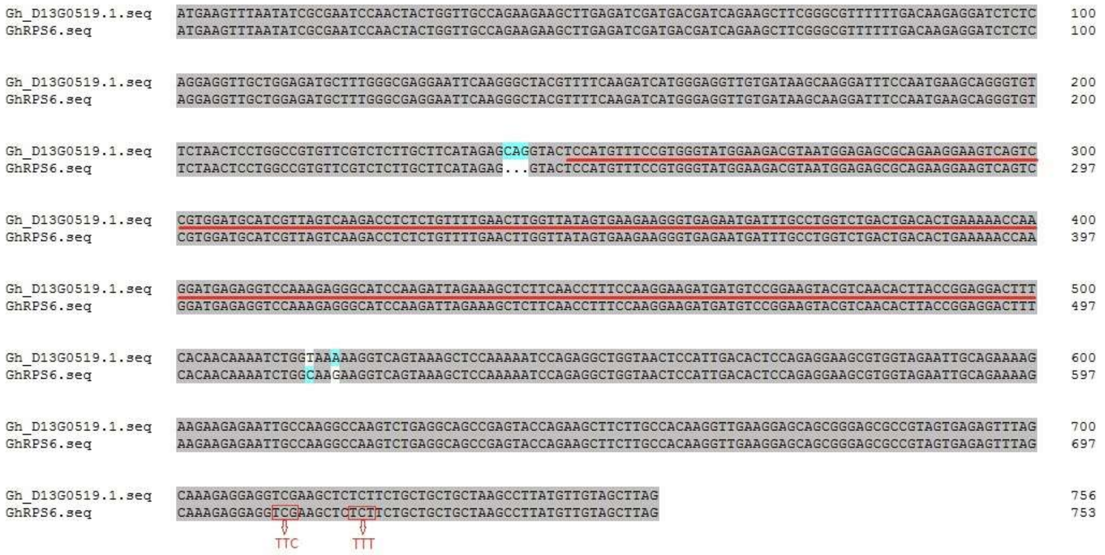

Supplementary Figure S2. The *GhRPS6* sequence cloned from cDNA in the root of cotton cv zhongzhimian2 was compared with the sequence of Gh_D13G0519.1 in NAU Assembly. The red line is VIGS silencing fragment, the gray part is consistent sequence, and the arrow part is phosphorylation site mutation.

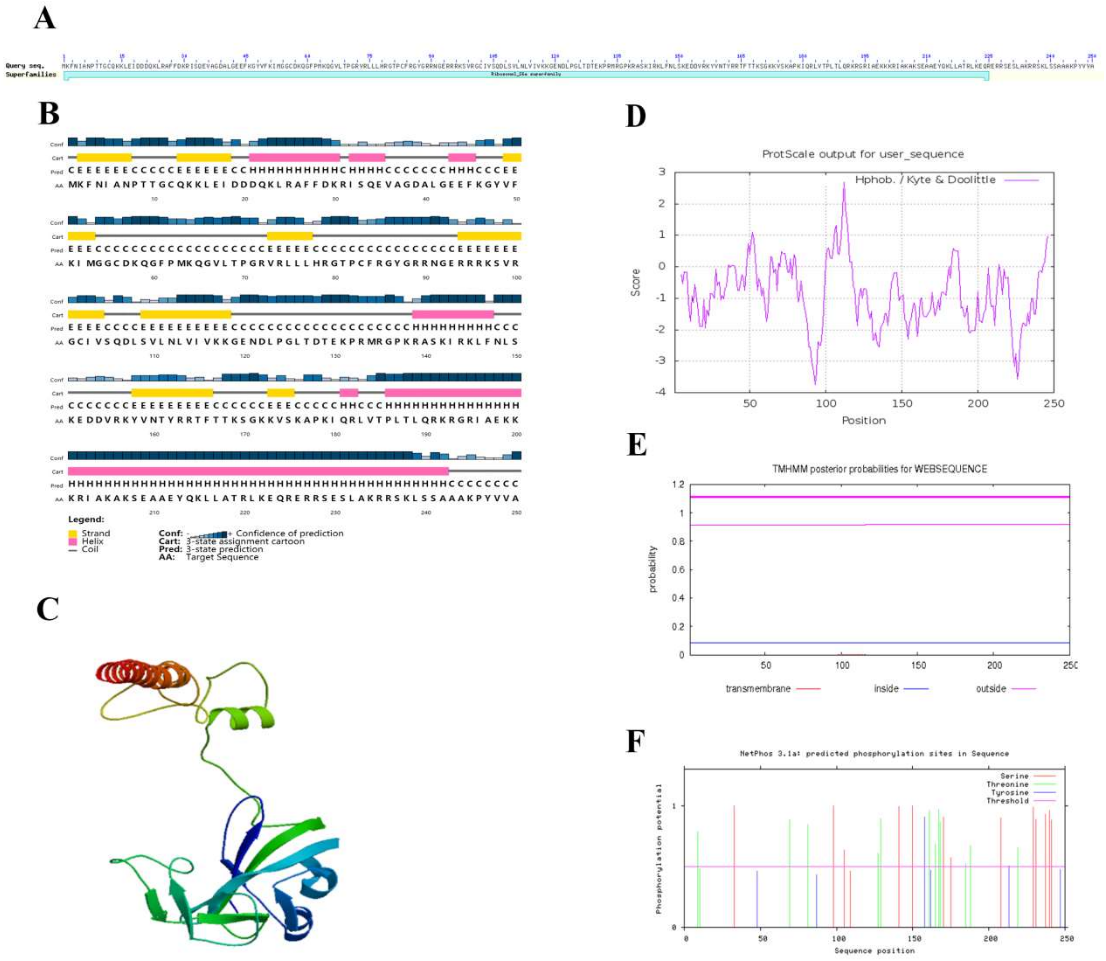

Supplementary Figure S3. Bioinformatics analysis of GhRPS6. A, The conserved domain of GhRPS6.B, Secondary domain of GhRPS6.C, The tertiary domain of GhRPS6.D, Hydrophilic and hydrophobic analysis of GhRPS6.E, Transmembrane domain analysis of GhRPS6.F, Phosphorylation sites of GhRPS6 protein were predicted. All amino acid sequences were translated by RT-PCR amplification of the roots of Zhongzhimian2.

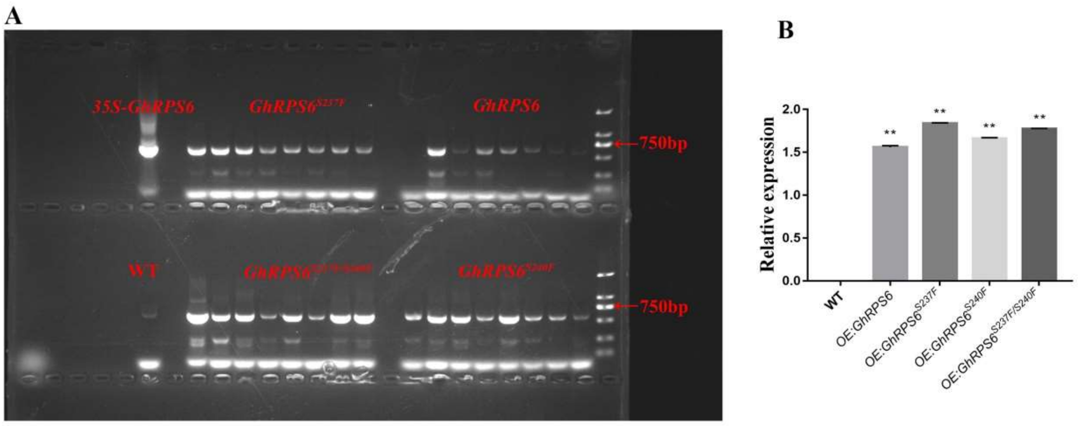

Supplementary Figure S4. Identification of transgenic *Arabidopsis thaliana*. A, PCR detection of transgenic *Arabidopsis thaliana*.B, Fluorescence quantitative analysis of GhRPS6 expression in *Arabidopsis thaliana*. Error bars represent the standard deviation of three biological replicates. Asterisks indicate statistically significant differences, as determined by Student's t-test (*P < 0.05; **P < 0.001).

Supplementary Tabel 1. Primers used in this research work.

**Primer name**	**Sequences(5′-3′)**	**Digestion sites**	**Destination**
GhRPS6-full-F	ATGAAGTTTAATATCGCGAATCCAAC		Gene cloning
GhRPS6-full-R	CTAAGCTACAACATAAGGCTTAGCAGCAG		
GhRPS6-VIGS-F	TCCATGTTTCCGTGGGTAT	Xba Ⅰ	VIGS
GhRPS6-VIGS-R	AAAGTCCTCCGGTAAGTGTTG	Sac Ⅰ	
GhRPS6-OE-F	CACGGGGGACTCTAGAATGAAGTTTAATAT CGCGAATCCAA	Xba Ⅰ	overexpression
GhRPS6-OE-R	CCTTTACCCATGTTAATTAAAGCTACAACAT AAGGCTTAGCAGC	Sac Ⅰ	
GhRPS6^{S237F}-F	GTTTAGCAAAGAGGAGGTTCAAGCTCTCTT		237 Ser→Phe
GhRPS6^{S237F}-R	GAACCTCCTCTTTGCTAAACTCTCACTACG		
GhRPS6^{S240F}-F	AGAGGAGGTCGAAGCTCTTTTCTGCTGCTG		240 Ser→Phe
GhRPS6^{S240F}-R	AAGAGCTTCGACCTCCTCTTTGCTAAACTC		
GhCHI-F	AATGACACGGCGACTCCCTT		qRT-PCR for *GhCHI*
GhCHI-R	TCCCACGAACCCCCACCTAT		
GhPAL-F	TGGTGGCTGAGTTTAGGAAA		qRT-PCR for *GhPAL*
GhPAL-R	TGAGTGAGGCAATGTGTGA		
GhPPO-F	CCGCATAACCATCACAAG		qRT-PCR for *GhPPO*
GhPPO-R	ACTCTCATCACCTTCAACA		
GhJaz1 -F	AGCCTCAAAAAGGAAGACCTCAAAC		qRT-PCR for *GhJaz1*
GhJaz1 -R	TGGCTGCTCAATCACCATAGTAATC		
GhNOA -F	GAGGATGCTGAAAGACCTGCTA		qRT-PCR for *GhNOA*
GhNOA -R	TCTCAACTGGCTTGGGTACATG		
GhC4H1 -F	CCGAACCCGACACCCATAAGC		qRT-PCR for *GhC4H1*
GhC4H1 -R	GCAGGGATGTCATACCCACCAAG		
GhUbiquitin-F	GAGTCTTCGGACACCATTG		qRT-PCR for endogenous reference gene of *Gossypium hirsutum*
GhUbiquitin-R	CTTGACCTTCTTCTTCTTGTGC		
GhRPS6 -R	GACGCACAATCCCACTATCC		PCR detection of transgenic *Arabidopsis thaliana*
GhRPS6 -R	CTAAGCTACAACATAAGGCTTAGCAGCAG		
